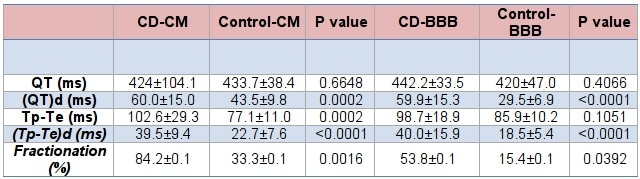# Repolarization Parameters Are Associated With Mortality In Chagas Disease Patients In The United States

**DOI:** 10.1016/s0972-6292(16)30773-2

**Published:** 2014-07-15

**Authors:** Jason Bradfield, Brandon Woodbury, Mahmoud Traina, Salvador Hernandez, Daniel Sanchez, Robin Wachsner, Kalyanam Shivkumar, Sheba Meymandi

**Affiliations:** 1 Center of Excellence for the Treatment of Chagas Disease, Olive View-UCLA Medical Center, Sylmar, CA; 2 UCLA Cardiac Arrhythmia Center, Ronald Reagan UCLA Medical Center, David Geffen School of Medicine at UCLA, Los Angeles, CA

**Keywords:** Chagas Disease, Repolarization Parameters, Mortality

## Abstract

**Objective:**

The goal of this study was to examine the association between ECG repolarization parameters and mortality in Chagas disease (CD) patients living in the United States.

**Methods:**

CD patients with cardiomyopathy (CM) and bundle branch block (BBB) or BBB alone were compared to age- and sex-matched controls. QT interval, QT dispersion (QTd), T wave peak to T wave end duration (Tp-Te) and T wave peak to T wave end dispersion ((Tp-Te)d) were measured. Presence of fractionated QRS (fQRS) was also assessed. The main outcome measure was the association between ECG parameters and mortality or need for cardiac transplant.

**Results:**

A total of 18 CM and 13 BBB CD patients were studied with 97% originating from Mexico or Central America. QTd (60.0±15.0 ms vs 43.5±9.8 ms, P=0.0002), Tp-Te (102.6±29.3 ms vs 77.1±11.0 ms, P=0.0002) and (Tp-Te)d (39.5±9.4 ms vs 22.7±7.6 ms, P<0.0001) were prolonged in CD CM patients compared to CM controls. Chagas CM patients had more fQRS then controls (84.2±0.10% vs 33.3±0.11%, p=0.0005). QTd (59.9±15.0 ms vs 29.5±6.9 ms, P=0.0001) and (Tp-Te)d (40.0±15.9 ms vs 18.5±5.4 ms, p<0.0001) were longer in the CD BBB group compared to BBB controls. Univariate analysis showed QTd (56.9±15.0 ms vs 46.5±17.3 ms, p=0.0412) and (Tp-Te)d (36.8±13.5 ms vs 28.5±13.3 ms, p=0.0395) were associated with death and/or need for cardiac transplant.

**Conclusion:**

Our results indicate that P-max and PD are useful electrocardiographic markers for identifying the β-TM-high-risk patients for AF onset, even when the cardiac function is conserved.

## Introduction

Sudden cardiac death (SCD) is the most common cause of mortality in Chagas disease (CD) patients from South America and CD patients with cardiomyopathy (CM) are known to have a higher mortality than patients with non-ischemic cardiomyopathy (NICM) not related to CD.[1] Further, CD patients, even without CM, living in South America with cardiac involvement are at increased risk for SCD.[2] SCD is estimated to be the cause of mortality in approximately 47.6% of all CD patient deaths.[3] Repolarization parameters have been shown to be associated with this increased mortality [4] in a South American population.

However, less is known about SCD risk and clinical outcomes for CD patients living in the United States (US).[5] Numerous vectors for the transmission of CD exist in the US, but very few cases of autochthonous transmission within the country have been documented [6] and the majority of patients with CD in the US contracted the disease while living in endemic countries prior to immigration to the US.[7]

One reason this population living in the US requires further study is that much of previous research on Chagas heart disease involves patients from the Southern Cone region of South America where the T Cruzi II strain predominates.[8] However, the majority of patients in the US are immigrants from Mexico and Central America, where T. Cruzi I strain is primarily found.[9-12] There is limited clinical data on risk stratification of this subset of CD patients. It is possible that strain differences between regions may lead to differences in the severity of cardiac involvement and variability in SCD risk.[8,13,14]

New and important clinical data is beginning to emerge regarding CD patients living in the US. Recent data from our institution suggests that the prevalence of CD in areas with large Latin American immigrant populations is higher than expected in a community screening program at 1% [15], while the prevalence was 5.3% [16] when patients from endemic regions with bundle branch block (BBB) are screened and 19% in the CM patient population from endemic regions [16]. In addition our group has recently presented data that the overall mortality is higher in the CD CM population (13.4%) than in NICM patients (2.9%) with other etiologies of CM.[17]

In the current study we evaluated depolarization and repolarization parameters in a population of CD patients with CM (CD-CM) and BBB (CD-BBB) from endemic regions cared for at our institution. We compared these patients to a group of age- and sex-matched control patients from endemic regions without CD who have CM (control-CM) and/or BBB (control-BBB), and assessed the relationship of these parameters to death or need for cardiac transplant.

## Methods

All CD patients with CM and BBB (n = 18) or BBB alone with a preserved ejection fraction (EF) (n = 13) followed at the Olive View-UCLA Center of Excellence for the Treatment of CD with at least one year of available follow-up were included. BBB was defined as right bundle branch block (RBBB) and/or left anterior fascicular block (LAFB). Age- and sex-matched controls were obtained in a 1:1 ratio. The controls were taken from a group of patients born in endemic CD regions who had similar findings of CM and/or BBB and were screened for CD but found to have negative titers. CD positivity was verified with testing utilizing immumofluorescent assays and enzyme-linked immunosorbent assays through the Centers for Disease Control and Prevention. All patients labeled as having CM secondary to CD were previously found to have no significant coronary artery disease (CAD) based on previous coronary angiography or stress testing and had no history consistent with an alternative etiology for NICM.

Standard 12-lead ECGs were reviewed by two physicians blinded to patient groups and classified based on the presence or absence of a fractionated QRS (fQRS) on resting ECG. Fractionation was determined based on the work of Das and colleagues [18-20] and defined as: 1) notching in the nadir of the R or S wave or >1 R' in two contiguous leads in a coronary artery territory distribution for a narrow QRS complex; or 2) in the setting of BBB >2 notches were required in the R wave or S wave; or 3) for a PVC beat >2 peaks or notches were required in 2 contiguous leads or two notches in the R wave greater than 40 ms apart.

Repolarization parameters including QT interval, QT dispersion (QTd), T wave peak to T wave end duration (Tp-Te) and T wave peak to T wave end dispersion ((Tp-Te)d) were measured using electronic calipers (Philips TraceMasterVue ECG Management System, Philips Healthcare, Andover, MA) in each patient. QT interval was measured from the beginning of the QRS complex to the end of the T wave, defined as the tangent to the downslope of the T wave and the isoelectric line.[21] QTd was defined as the difference between the maximum and minimum QT interval on all 12-leads of the ECG. Tp-Te was obtained from the difference between the QT interval and the QT peak interval, which was measured from the beginning of the QRS to the peak of the T wave. For a negative or biphasic T wave, the QT peak was measured to the nadir of the T wave. (Tp-Te)d was obtained by the difference between the maximum and minimum Tp-Te interval in the precordial leads.[21]

Follow-up data and mortality rates were based on review of electronic medical records, direct phone call follow-up with patients and/or available family contacts and review of the social security database. Findings in the CD-CM and CD-BBB were compared to the matched controls and to each other.

### Statistical analysis

Continuous variables are expressed as mean ± SD. Statistical significance was assessed using one way analysis of variance after examining normal quantile plots to determine that the values followed the normal distribution. Categorical variables, expressed as frequencies and percentages, were compared using Fisher's exact test. No adjustments were made for multiple comparisons, as this is an exploratory study.

## Results

There was no significant difference in baseline characteristics between CD-CM or CD-BBB groups and their age/sex matched controls. (Table 1) Mean age (years) in the CD-CM group was 61±14 vs 62±13 in the control-CM group (p=0.8428) and mean age in the CD-BBB group was 50±11 vs 50±11 in the control-BBB group (p=1.0). Patients had similar baseline left ventricular EF (CD-CM 24.5±8.6 % vs control-CM 27.7±10.0 %, p=0.3106; CD-BBB 59.8±5.2 % vs control-BBB 59.2±4.9 %, p=0.7647). Ninety-seven percent of the patients in the CD and control groups were from Mexico or Central America, with the largest representation from Central America originating from El Salvador and Guatemala. One patient each in the CD-BBB and control-BBB group were from South America (Argentina and Peru respectively). A similar percentage of patients were on appropriate CM medications (beta blockers and angiotensin-converting enzyme inhibitors/angiotensin receptor blockers) that have the potential to affect mortality. In addition similar numbers of patients in the CD and control groups had implantable cardioverter-defibrillators (ICDs) in place. QRS duration was similar between the BBB groups (120±25 ms in the CD-BBB vs 116±24 ms in the control-BBB) and the CM groups (130±31 ms in the CD-CM vs 139±36 ms in the control-CM). There was no difference in the use of amiodarone between the BBB groups (1/13 (8%) in CD-BBB vs 0/13(0%) in control-BBB, p=1.0). There was a trend toward increased amiodarone use in the CD-CM group [5/18 (28%) vs 1/18 (6%)] compared to the control-CM group, but it did not reach statistical significance (p=0.18). The average follow-up was similar between groups [1976±748 days in the CD patients (1933±254 days in CD-BBB; 2015±1028 days in CD-CM) vs 1890±846 days in the control groups (1826±636 days in control-BBB; 1938±875 days in control-CM), p=0.7822].

When comparing the CD-CM with the CD-BBB groups there was a significant difference in age with CD-CM patients being older (60.8±14.1 years vs 49.8±11.4 years, p=0.019). As expected the CD-CM patients had a lower left ventricular EF (24.5±8.6 % vs 59.8±5.2 %, p=0.0001). More fractionation of the QRS was seen in the CD-CM group than the CD-BBB group (84.2% vs 53.8%, p=0.00012). However, there was no significant difference in QT, QTd, Tp-Te or (Tp-Te)d.

CD-CM patients had more fQRS than control-CM patients (84.2±0.10% vs 33.3±0.11%, p=0.0005). The CD-CM QT (424.2±104.1 ms vs 433.72±38.4 ms, p=0.66) was not statistically different than controls, however the QTd (60.0±15.0 ms vs 43.5±9.8 ms, P=0.0002), Tp-Te (102.6±29.3 ms vs 77.1±11.0 ms, P=0.0002) and (Tp-Te)d (39.5±9.4 ms vs 22.7±7.6 ms, P<0.0001) were prolonged compared to controls. (Table 2)

CD-BBB patients had prolonged QTd (59.9±15.0 ms vs 29.5±6.9 ms, P=0.0001) and (Tp-Te)d (40.0±15.9 ms vs 18.5±5.4 ms, p<0.0001) compared to control-BBB patients. (Table 2) In addition, CD-BBB patients were found to have increased QTd (59.9±15.0 ms vs 43.5±9.8 ms, P=0.0006), Tp-Te (98.7±18.9 ms vs 77.1±11.0 ms, P=0.004) and (Tp-Te)d (40.0±15.9 ms vs 22.7±7.6 ms, P=<0.0001) compared to the control-CM patients.

**Figure 1:**
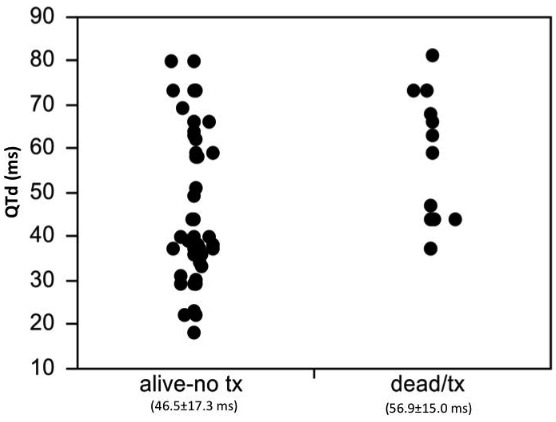
Jitter-plot of QTd measurement comparing surviving patients to those that died or underwent cardiac transplant. ms= milliseconds, tx=transplant.

At the time of analysis, after a mean follow-up of 1976±748 days in the CD patients vs 1890±846 days in the control groups (p=0.7822), 11 patients with CD (10 CM and 1 BBB) had died and 1 received a cardiac transplant. Three patients in the control-CM and no control-BBB group patients had died. The mortality was significantly higher in the CD-CM group compared to the CM-control group (56% vs 17%, p=0.022) Univariate analysis demonstrated that QTd (56.9±15.0 ms vs 46.5±17.3 ms, p=0.0412), (Tp-Te)d (36.8±13.5 ms vs 28.5±13.3 ms, p=0.0395) and left ventricular EF (26±13% vs 44±17%, p=0.001) were associated with death or transplant. (Figure 1)

## Discussion

CD, caused by the protozoan Trypanosoma Cruzi (T. Cruzi) was previously estimated by the World Health Organization to affect some 16-18 million people throughout the American continent [22], including over 300,000 people in the US based on a 2009 estimate.[23] The worldwide estimate has decreased in recent years due to public health initiatives with a recent estimate of approximately 8 million infected people.[24] However, while public health initiatives in Southern and Central America have decreased the incidence of new infections [25], CD remains a significant cause of cardiac morbidity and mortality.

Heart disease is the most frequent clinical manifestation of CD, affecting approximately 30% of infected individuals after twenty years of initial exposure. [2,26] Chagas heart disease is characterized by cardiomegaly, congestive heart failure, and rhythm disturbances including conduction system disease, ventricular arrhythmias, and SCD.[2,27,28] Conduction system disturbances, most commonly RBBB and LAFB precede the development of CM by many years. However there appears to be a significantly increased mortality in this population even prior to the development of CM.[2]

The increased SCD rate in the CD population has not been completely explained in the literature. In addition to the structural abnormalities in the CM population there is also a potential autonomic component due to destruction of parasympathetic innervation [29] that may lead to a mismatch of sympathetic and parasympathetic stimulation.

It is known in the non-CD population that increased sympathetic cardiac stimulation can increase the risk of malignant ventricular arrhythmias.[30,31] One mechanism that may contribute to this increased risk is more pronounced dispersion of repolarization and risk for induction of reentrant ventricular arrhythmias. Recent studies have shown that neuraxial modulation via thoracic epidural anesthesia or surgical left cardiac sympathetic denervation can significantly decrease arrhythmia burden in a non-CD CM population.[32] However, this potential contribution to, and intervention for, SCD has not been proven in the CD population.[29]

The use of surface 12-lead ECG intervals as surrogate evidence of dispersion of repolarization to predict risk of SCD dates back to the work of Schwartz and colleagues.[33] QTd has been studied extensively and been shown to be a marker of risk for ventricular arrhythmias after myocardial infarction [34] and in patient with depressed left ventricular EFs [35]. QTd has been evaluated as a risk marker in CD as well and is an independent predictor of all-cause death and arrhythmic death in CD patients in South America.[4] Other repolarization parameters such as Tp-Te and (Tp-Te)d have been studied in other populations such as hypertrophic cardiomyopathy [36] and Brugada syndrome [21] with evidence that these parameters may be a better predictor than QTd for inducible ventricular arrhythmias, spontaneous ventricular arrhythmias, need for appropriate ICD therapy [37] and SCD.[38] The depolarization parameter, fragmented QRS (fQRS), is a known marker of increased mortality in CAD [19] and a marker of increased arrhythmic events in CAD, ischemic CM and NICM [39,40] as well as Brugada syndrome [41], but has not been previously studied in a CD population. These parameters need further data in CD.

In this study, CD patients living in the US with CM and BBB or BBB with preserved EF have increased evidence of dispersion of repolarization measured by QTd and (Tp-Te)d compared to age- and sex-matched controls from endemic regions. CD-CM patients could only be differentiated from CD-BBB patients based on increased frequency of fQRS, but not on repolarization parameters. The increased QTd and (Tp-Te)d was associated with increased mortality and/or need for transplant. These findings may give insight into the increased mortality risk in this patient population.

Interestingly, there was no difference in QTd or (Tp-Te)d between the CD-CM and CD-BBB group suggesting that the pathologic changes that may predispose to ventricular arrhythmias and SCD may precede the development of frank left ventricular dysfunction. Further data is needed to determine if CD patients with BBB, but without CM, originating from Central America and Mexico have a significant mortality risk that suggest the need for more aggressive intervention such as ICD implantation prior to the development of a depressed EF.

Further, CD-BBB patients had more significant QTd and (Tp-Te)d than the control-CM group. This may call into question the current guidelines which advocate specific therapies based predominantly on discrete EF criteria. The current criteria may not accurately predict risk in the CD patient population.

## Limitations

The limited sample size of our study makes multivariate analysis impossible which limits the interpretability of the study. Conclusions based on associations of mortality are also limited by the lack of documented evidence of SCD as the etiology of death.

Similar repolarization parameters were found when comparing CD-CM and CD-BBB patients. The majority of deaths occurred in the CD-CM group however. Therefore, an argument can be made that repolarization abnormalities alone are not sufficient to increase the risk of death in the CD population. However, the number and length of follow-up of CD-BBB patients was limited and therefore further data is needed.

## Conclusion

Over 20 years has passed since the first publication on Chagas heart disease in the US by Hagar and Ramtoola in 1991 [5] with limited assessment of this patient population over that timespan. This is to date the largest study of chagas heart disease patients in the US and the only study on a US population assessing predictors of mortality. QTd and (Tp-Te)d were prolonged in CD-CM and CD-BBB patients compared to controls. Measures of dispersion were also significantly prolonged in the CD-BBB in comparison to CM controls. Univariate analysis demonstrated that QTd and (Tp-T)d were associated with death or transplant in the CD population. Further data is needed to assess the significance of these findings and to determine if region of origin and strain of T. cruzi infection affect clinical outcomes.

## Figures and Tables

**Table 1:**
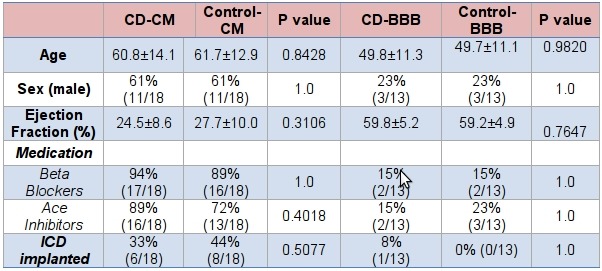


**Table 2:**