# Repolarization Parameters Are Associated With Mortality In Chagas Disease Patients In The United States

**DOI:** 10.1016/s0972-6292(16)30802-6

**Published:** 2014-10-06

Conclusion of the abstract has been corrected as: CD patients with CM and BBB or BBB alone have increased evidence of dispersion of repolarization compared to controls. QTd and (Tp-Te)d were associated with increased mortality and/or need for transplant.

